# Time From Anaesthetic Start to Surgery Start in Hysteroscopy and Theatre List Overrun in Gynaecology: A Retrospective Observational Study

**DOI:** 10.7759/cureus.119211

**Published:** 2026-11-06

**Authors:** Fadi Alhusban, Yaman Al Ajlouni, Alistair Bashall, Laura Clabon, Camilla Roche

**Affiliations:** 1 Anaesthesiology and Critical Care, University Hospital Lewisham, London, GBR; 2 Critical Care Medicine, Lewisham and Greenwich NHS Trust, London, GBR; 3 Anaesthesia, Lewisham and Greenwich NHS Trust, London, GBR

**Keywords:** anaesthetic preparation time, body mass index, gynaecological surgery, hysteroscopy, operating theatre efficiency, perioperative management, theatre list overrun, theatre scheduling

## Abstract

Background

We evaluated patient-level factors associated with the interval from anaesthetic start to surgery start in hysteroscopy and list-level factors associated with overrun in elective gynaecology lists.

Methods

This retrospective observational study used 2025 nursing pre-assessment and theatre timing records from two hospitals within one National Health Service (NHS) trust. Duration was analysed using a lognormal mixed model with a theatre-list random intercept, adjusting for recorded pre-assessment characteristics, session, site, and mixed-list status. Body mass index (BMI) was modelled using a four-knot restricted cubic spline. Adjusted mean differences were reported in minutes. Secondary analyses used mixed-effects logistic regression for intervals ≥22 minutes and multivariable logistic regression for list overrun, defined as last-patient departure after the scheduled finish. Sensitivity analyses examined untransformed duration and excluded all-day lists.

Results

Among 913 hysteroscopies, 890 cases across 491 theatre lists were included in adjusted analyses. BMI was nonlinearly associated with duration (overall p<0.001; nonlinearity p=0.007). Compared with BMI 30 kg/m², adjusted mean intervals were 1.42 minutes longer (95% confidence interval (CI) 0.46-2.43) at 40 kg/m² and 4.67 minutes longer (95% CI 2.56-6.90) at 50 kg/m². These contrasts were similar in sensitivity analyses. Among 611 gynaecology lists, 302 (49.4%) overran. Lists combining minor and major procedures had higher odds of overrun than lists containing only minor procedures (adjusted odds ratio (OR) 3.10; 95% CI 2.00-4.80). Afternoon lists had lower odds than morning lists (OR 0.23; 95% CI 0.15-0.34), while odds were higher at University Hospital Lewisham than Queen Elizabeth Hospital (OR 2.18; 95% CI 1.49-3.20). Lists in which at least half of minor cases had prolonged intervals also had higher odds of overrun (OR 1.52; 95% CI 1.04-2.23), but evidence for this association was less certain after excluding all-day lists (OR 1.40; 95% CI 0.92-2.11).

Conclusions

Higher BMI was associated with longer intervals from anaesthetic start to surgery start in hysteroscopy. Lists combining minor and major procedures had higher odds of overrun. Prospective studies should evaluate whether accounting for preparation requirements and list composition improves scheduling. These findings do not establish a BMI threshold or booking rule.

## Introduction

Operating theatre efficiency is an important determinant of health-system performance, particularly in publicly funded services such as the National Health Service (NHS). Operating theatres are among the most resource-intensive areas of secondary care, and their efficiency can be influenced by modifiable operational factors [1-3].

Improving theatre efficiency is an NHS priority given its potential to reduce waiting lists and preserve capacity for urgent and time-sensitive care [1,4]. Advances in surgical and anaesthetic practice have enabled more procedures to be performed as day cases, increasing the importance of efficient theatre use [5].

Theatre efficiency can be assessed using on-time starts, theatre utilisation, cancellations, turnover time, list overruns or underruns, and patient waiting time; however, the comparative value of individual metrics and composite scores is uncertain [2,6]. Research into theatre inefficiency has frequently focused on cancellations and their potentially preventable patient-, hospital-, and preparation-related causes [7-10]. Elective list overruns can contribute to cancellations, reduced throughput, overtime costs, and poor patient experience [3,10]. Although overruns have been associated with patient, staffing, scheduling, and workflow factors, the relative contributions of case number and case complexity remain uncertain [2,3].

On high-volume lists of short procedures, such as elective hysteroscopy lists, small delays in anaesthetic induction, surgery, recovery, or turnover can accumulate across successive cases and delay list completion [6,11]. Understanding these delays requires consideration of patient-level preparation and list-level organisation rather than reliance on a single measure such as case number. We aimed to identify patient-level factors associated with the interval from anaesthetic start to surgery start in hysteroscopy and list-level factors associated with overrun in elective gynaecology lists containing at least one minor procedure.

## Materials and methods

Ethical considerations

This study received Health Research Authority (HRA) and Health and Care Research Wales (HCRW) approval on 6 May 2026 (approval number: 26/HRA/1725; IRAS project ID: 370948). NHS Research Ethics Committee review was not required, as documented in the approved study protocol. Lewisham and Greenwich NHS Trust acted as the study sponsor. Reporting followed the Strengthening the Reporting of Observational Studies in Epidemiology (STROBE) statement and its REporting of studies Conducted using Observational Routinely-collected health Data (RECORD) extension [12,13].

Study design and setting

This retrospective observational study evaluated elective gynaecology activity at Queen Elizabeth Hospital (QEH) and University Hospital Lewisham (UHL), within Lewisham and Greenwich NHS Trust, from 1 January to 31 December 2025. Patient-level analyses examined the interval from anaesthetic start to surgery start in hysteroscopy. List-level analyses examined overrun in elective gynaecology lists containing at least one minor procedure.

Study population

Patient-Level Cohort

The patient-level cohort comprised 913 patients undergoing hysteroscopy, irrespective of anaesthetic technique. All patients were recorded as female in the source data. Each patient contributed one hysteroscopy case, although individual theatre lists could contain multiple hysteroscopies. Hysteroscopy was selected as a high-volume minor procedure with a relatively consistent perioperative workflow. All 913 cases were retained in descriptive summaries; 890 cases with complete data for the model variables were included in the main adjusted analyses.

List-Level Sample

The list-level sample comprised 611 elective gynaecology lists containing at least one minor procedure, with each list treated as a single analytical unit. Lists without minor procedures were outside the extraction criteria. Lists were excluded when surgical issues during the first minor case occupied almost the entire session and prevented the next patient from being called. This exclusion criterion was defined before examining the original list-overrun results. Eligible hysteroscopy cases from these excluded lists remained in the patient-level cohort. All available records meeting the respective cohort criteria during 2025 were included; no formal sample size calculation was performed.

Data sources and variables

Data were extracted from routine theatre and perioperative records using a structured template. Pre-assessment characteristics were obtained exclusively from nursing pre-assessment records and were coded as present only when explicitly documented. Patient-level model variables included age, body mass index (BMI), American Society of Anesthesiologists (ASA) physical status, documented obstructive sleep apnoea or severe respiratory disease, documented history of difficult airway, severe anxiety or needle phobia, interpreter requirement, session, site, and mixed-list status.

Severe respiratory disease was defined as a previous respiratory-related critical care admission or documented inability to complete a six-minute walk test. A positive difficult-airway history required explicit documentation of a previous difficult airway by a doctor, as recorded in the nursing pre-assessment record; difficulty was not inferred from other observations. For binary pre-assessment characteristics, undocumented findings were coded 0, so this category included both documented absence and non-documentation. Aspiration risk and the flag for additional anaesthetic time were not modelled because documentation was sparse. List-level variables included list composition, case number, session, site, and observed prolonged-interval burden among minor cases. Data were pseudonymised and handled in accordance with NHS information governance and institutional data-protection requirements.

Data were extracted from two routine electronic systems: nursing pre-assessment records in iCare and theatre scheduling and timing records in BI Hub. Source timestamps ("anaesthetic start", "surgery start", and "patient out of theatre") were entered manually by a designated theatre team member, usually a nurse, as the events occurred. Hysteroscopy cases were identified by filtering recorded procedure names for "hysteroscopy". Eligible lists were identified by filtering for gynaecology specialty, elective status, study period, and the presence of at least one minor procedure. Booked total theatre durations were based on standard procedure templates. Procedures booked for ≤45 minutes were classified as minor, whereas those booked for >45 minutes were classified as major, with no intermediate category.

BI Hub and iCare records were linked using unique hospital numbers. After data collection and record linkage were complete, hospital numbers were removed from the analysis dataset as part of pseudonymisation. Patients were grouped into theatre lists using date, site, theatre, and session. These list links were provisional. Patients from lists excluded from the list-level analysis retained their original list identifiers for the patient-level analysis. Data checks covered missing values, unusual values, inconsistent time intervals, and duplicate records. No cases were excluded solely because of extreme durations or their influence on the model. All 913 hysteroscopy cases had duration data and were included in descriptive summaries. The main adjusted analyses included 890 cases with complete data for the model variables. No data were imputed.

Outcome definitions

The primary patient-level outcome was the elapsed time, in minutes, from recorded anaesthetic start to recorded surgery start. Anaesthetic start was recorded after completion of the World Health Organization (WHO) checklist Sign In and attachment of patient monitors. Surgery start was recorded after WHO Time Out, positioning, and draping, when the surgeon asked to begin and the team confirmed readiness. Anaesthetic preparation took place in the anaesthetic room before transfer to theatre. The measured interval therefore included anaesthetic preparation, transfer, and preparation for surgery.

A prolonged interval, defined as ≥22 minutes, corresponding to the cohort’s 75th percentile, was retained as a secondary patient-level outcome. The list-level outcome was overrun, defined as the last patient leaving theatre after the scheduled list finish: 12:30 for morning lists and 17:30 for afternoon and all-day lists.

Exposure definitions

A mixed list contained both minor and major gynaecological procedures, whereas a non-mixed list contained only minor procedures. The proportion of minor cases with an interval ≥22 minutes was calculated for each list, including all eligible minor gynaecological procedures. Lists in which at least half of minor cases met this threshold were classified as having a high prolonged-interval burden. This measure was calculated retrospectively from recorded timings and was not available when lists were scheduled. Total cases per list was analysed as a continuous variable.

Missing data

The main adjusted patient-level analyses included 890 of 913 cases (97.5%) with complete data for all model variables. The remaining 23 cases (2.5%) were retained in descriptive summaries but excluded from these adjusted analyses. All model variables were complete for the 611 lists included in the list-level analyses.

Statistical analysis

Continuous data are reported as medians (interquartile range (IQR)) or as means (standard deviation (SD)) and range where specified. Categorical data are reported as counts (percentages).

The continuous primary analysis was introduced after the original binary analyses. We fitted a linear mixed model to the natural logarithm of duration, with a random intercept for theatre list to account for patients sharing the same list. This lognormal model was selected after examining residual plots from models of untransformed and log-transformed duration. All patient-level covariates described above were entered together and retained regardless of their individual statistical significance. Age was modelled linearly, with effects reported per 10-year increase. BMI was modelled using a four-knot restricted cubic spline, with knots at the 5th, 35th, 65th, and 95th percentiles of the complete-case BMI distribution (21.4635, 27.30, 33.2955, and 47.00 kg/m²). ASA physical status, site, session, and binary characteristics were treated as categorical variables. Model parameters were estimated using restricted maximum likelihood (REML).

Overall BMI association was assessed by jointly testing all three BMI terms, while nonlinearity was assessed by testing the two nonlinear terms. These likelihood-ratio tests used three and two degrees of freedom, respectively. Comparisons used maximum-likelihood estimation, with the same patients, other covariates, and list random-intercept structure. We estimated adjusted mean durations across the observed BMI range and compared BMI values of 40, 45, and 50 kg/m² with 30 kg/m². At each BMI value, predictions were averaged across the 890 patients, keeping their other covariates at their observed values. Predictions were converted to arithmetic means in minutes using both the between-list and residual variances. We reported adjusted mean differences in minutes and time ratios. Pointwise 95% confidence intervals (CIs) were obtained from 5,000 parametric bootstrap refits. Each replicate simulated new list effects and residuals, retained the observed covariates and list sizes, and refitted all model parameters. Confidence limits were the 2.5th and 97.5th percentiles of the bootstrap estimates.

The secondary patient-level outcome, an interval ≥22 minutes, was analysed using mixed-effects logistic regression with a theatre-list random intercept. The model used the same covariates and BMI spline specification as the primary analysis. It was fitted by maximum likelihood using 121-point Gauss-Hermite quadrature. Adjusted odds ratios (ORs) and 95% Wald CIs were reported. Overall BMI association and nonlinearity were assessed using joint likelihood-ratio tests. BMI contrasts compared values of 40, 45, and 50 kg/m² with 30 kg/m².

Two sensitivity analyses assessed the primary duration findings. First, we fitted a linear mixed model to untransformed duration in minutes, using the same covariates, BMI spline knots, and theatre-list random intercept. Second, we repeated the lognormal model after excluding patients linked to confirmed all-day lists. This addressed uncertainty about the recorded morning/afternoon categories for patients on those lists. Patients with unknown list-session status were retained. Adjusted predictions from both sensitivity models were averaged over the covariate distribution of the same 890 patients used in the primary analysis. Confidence intervals were obtained from 5,000 parametric bootstrap refits for each model. Checks of the primary lognormal model included residual plots, convergence assessment, and refitting after omitting each patient and each theatre list in turn.

List overrun was analysed using multivariable logistic regression in 611 lists, with one observation per list. The model included mixed-list status, high prolonged-interval burden, total number of cases, session (morning, afternoon, or all-day), and hospital site. Case number was entered as a linear term, and the other predictors were categorical. Adjusted ORs and 95% Wald CIs were reported. A sensitivity analysis excluded the 69 all-day lists and repeated the model in the remaining 542 lists using the same predictors.

The list-level logistic models were compared with intercept-only models using likelihood-ratio tests. Model fit was summarised using McFadden's and Nagelkerke's pseudo-R² and discrimination using the area under the receiver operating characteristic curve (AUC). These measures were calculated in the same data used to fit the models. Statistical tests were two-sided, with a nominal significance level of 0.05. No adjustment for multiple comparisons was applied.

Initial mixed-model fits and residual plots were obtained using jamovi version 2.7 with the GAMLj module. The final model estimates, likelihood-ratio tests, adjusted predictions, and bootstrap confidence intervals were calculated using custom Python scripts (Python 3.12.14; NumPy 2.3.5; SciPy 1.17.0; pandas 2.2.3). Figures were produced using Matplotlib 3.10.8.

Data governance

The investigators did not have unrestricted access to the complete source databases. A restricted BI Hub extract was provided by the Trust information and theatre teams, and access to iCare records was limited to authorised Trust personnel.

## Results

Patient-level cohort

The cohort comprised 913 hysteroscopy cases. The median (IQR) interval from anaesthetic start to surgery start was 17 (14-22) minutes. The median age was 54 (47-61) years, and the median BMI was 30.0 (25.9-36.4) kg/m². A prolonged interval (≥22 minutes) occurred in 234 cases (25.6%) (Table 1).

**Table 1:** Characteristics of hysteroscopy cases, overall and by interval from anaesthetic start to surgery start Continuous summaries use available observations. Percentages use the full column totals, including observations with missing data. Binary characteristics are shown as the number recorded as present. Pre-assessment characteristics were obtained exclusively from nursing records; undocumented findings were coded 0. Difficult-airway history required explicit documentation of a previous difficult airway by a doctor, as recorded in the nursing pre-assessment record. Data were missing for age (n=1), BMI (n=15), ASA physical status (n=4), OSA or severe respiratory disease (n=1), difficult-airway history (n=1), severe anxiety or needle phobia (n=2), interpreter requirement (n=2), and mixed-list status (n=3). Duration, session, and site data were complete. The ≥22-min threshold defined the secondary prolonged-interval outcome. IQR: interquartile range; BMI: body mass index; ASA: American Society of Anesthesiologists; OSA: obstructive sleep apnoea; QEH: Queen Elizabeth Hospital; UHL: University Hospital Lewisham

Characteristic	Overall (n=913)	Interval <22 min (n=679)	Interval ≥22 min (n=234)
Interval (min), median (IQR)	17 (14-22)	15 (13-18)	26 (23-30)
Age (years), median (IQR)	54 (47-61)	54 (46-61)	55 (48-63)
BMI (kg/m²), median (IQR)	30.0 (25.9-36.4)	29.4 (25.7-35.2)	32.4 (27.0-40.3)
ASA physical status I, n (%)	59 (6.5)	47 (6.9)	12 (5.1)
ASA physical status II, n (%)	690 (75.6)	536 (78.9)	154 (65.8)
ASA physical status III, n (%)	154 (16.9)	91 (13.4)	63 (26.9)
ASA physical status IV, n (%)	6 (0.7)	2 (0.3)	4 (1.7)
OSA or severe respiratory disease, n (%)	59 (6.5)	34 (5.0)	25 (10.7)
Documented history of difficult airway, n (%)	39 (4.3)	28 (4.1)	11 (4.7)
Severe anxiety or needle phobia, n (%)	27 (3.0)	18 (2.7)	9 (3.8)
Interpreter required, n (%)	22 (2.4)	14 (2.1)	8 (3.4)
Morning session, n (%)	268 (29.4)	183 (27.0)	85 (36.3)
Afternoon session, n (%)	645 (70.6)	496 (73.0)	149 (63.7)
QEH, n (%)	550 (60.2)	410 (60.4)	140 (59.8)
UHL, n (%)	363 (39.8)	269 (39.6)	94 (40.2)
Mixed list, n (%)	467 (51.2)	338 (49.8)	129 (55.1)

Patient-level adjusted analyses

The primary analysis included 890 patients across 491 theatre lists. BMI was associated with the interval from anaesthetic start to surgery start (overall likelihood-ratio χ²=21.94; degrees of freedom (df)=3; p<0.001), with evidence of a nonlinear relationship (χ²=10.05; df=2; p=0.007). Compared with BMI 30 kg/m², adjusted mean intervals were 1.42 minutes longer (95% CI 0.46-2.43) at BMI 40 kg/m², 2.92 minutes longer (95% CI 1.55-4.30) at BMI 45 kg/m², and 4.67 minutes longer (95% CI 2.56-6.90) at BMI 50 kg/m² (Table 2; Figure 1).

**Table 2:** Adjusted associations with the interval from anaesthetic start to surgery start in hysteroscopy: primary lognormal mixed model Overall BMI association: likelihood-ratio χ²=21.94; df=3; p<0.001. BMI nonlinearity: likelihood-ratio χ²=10.05; df=2; p=0.007. The analysis included 890 patients across 491 theatre lists. A linear mixed model was fitted to the natural logarithm of duration, with a theatre-list random intercept. All covariates shown were included together, with BMI modelled using a four-knot restricted cubic spline. Estimates used restricted maximum likelihood; likelihood-ratio comparisons used maximum likelihood. Adjusted mean durations were standardised over the observed covariate distribution of the 890 patients and converted to arithmetic means using both between-list and residual variances. All 95% CIs were obtained from 5,000 parametric bootstrap refits and were pointwise, without adjustment for multiple comparisons. A time ratio above 1 indicates a longer interval and below 1 a shorter interval. For binary pre-assessment characteristics, the reference category combined documented absence and non-documentation. *The lower confidence limit for age was 0.99996 before rounding; the interval included 1. —: estimate not presented; CI: confidence interval; BMI: body mass index; ASA: American Society of Anesthesiologists; OSA: obstructive sleep apnoea; UHL: University Hospital Lewisham; QEH: Queen Elizabeth Hospital; df: degrees of freedom

Characteristic or comparison	Adjusted mean interval, min (95% CI)	Adjusted mean difference vs BMI 30, min (95% CI)	Adjusted time ratio (95% CI)
BMI 30 kg/m²	17.98 (17.34-18.68)	Reference	—
BMI 40 kg/m²	19.41 (18.57-20.32)	1.42 (0.46-2.43)	—
BMI 45 kg/m²	20.90 (19.78-22.10)	2.92 (1.55-4.30)	—
BMI 50 kg/m²	22.66 (20.75-24.74)	4.67 (2.56-6.90)	—
Age, per 10-year increase	—	—	1.018 (1.000-1.037*)
ASA II versus I	—	—	1.043 (0.954-1.139)
ASA III versus I	—	—	1.124 (1.006-1.256)
ASA IV versus I	—	—	0.944 (0.674-1.332)
OSA or severe respiratory disease, present versus reference	—	—	1.048 (0.954-1.152)
Documented history of difficult airway, present versus reference	—	—	0.970 (0.869-1.085)
Severe anxiety or needle phobia, present versus reference	—	—	1.071 (0.944-1.218)
Interpreter required, present versus reference	—	—	1.140 (0.984-1.319)
Mixed list versus list containing only minor procedures	—	—	1.022 (0.970-1.079)
Afternoon versus morning session	—	—	0.935 (0.885-0.990)
UHL versus QEH	—	—	0.996 (0.940-1.055)

**Figure 1:**
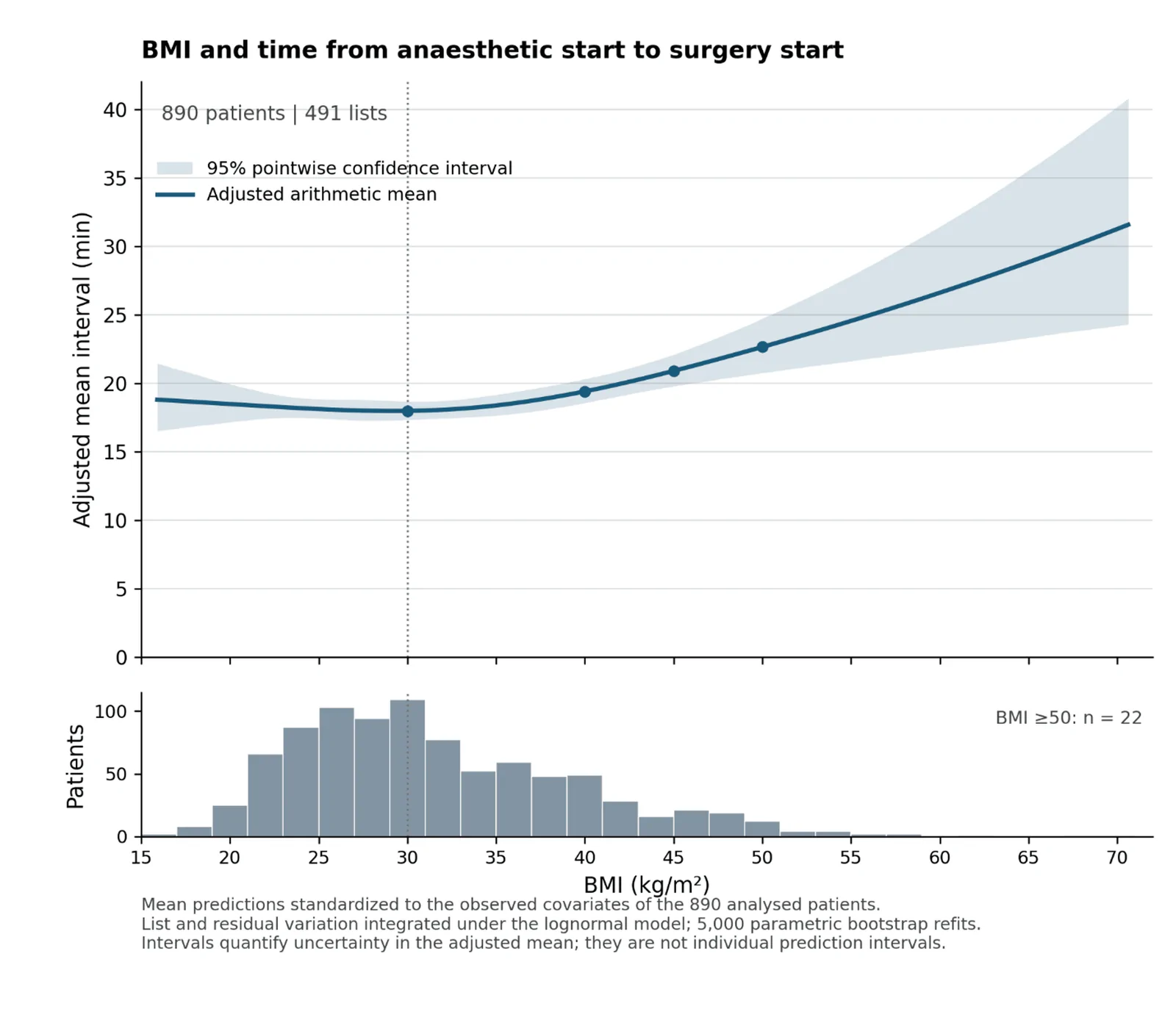
Adjusted association between BMI and the interval from anaesthetic start to surgery start (A) The solid curve shows the adjusted arithmetic mean interval from the primary lognormal mixed model, including 890 patients across 491 theatre lists. BMI was modelled using a four-knot restricted cubic spline. Predictions were standardised over the patients' observed covariates and incorporated between-list and residual variation. Shading represents pointwise 95% CIs from 5,000 parametric bootstrap refits. The vertical dotted line marks BMI 30 kg/m², the reference for the contrasts reported in Table 2. Circles mark estimates at BMI 30, 40, 45, and 50 kg/m². (B) The histogram shows the BMI distribution; 22 patients had BMI ≥50 kg/m². BMI: body mass index; CI: confidence interval

Afternoon sessions were associated with approximately 6.5% shorter intervals than morning sessions (adjusted time ratio 0.935; 95% CI 0.885-0.990). The estimated interval was 12.4% longer for ASA physical status III than for ASA physical status I (time ratio 1.124; 95% CI 1.006-1.256). Bootstrap 95% CIs included 1 for age, the other ASA comparisons, site, mixed-list status, documented history of difficult airway, obstructive sleep apnoea or severe respiratory disease, severe anxiety or needle phobia, and interpreter requirement (Table 2).

The BMI findings were similar in both sensitivity analyses. In the model of untransformed duration, adjusted mean differences compared with BMI 30 kg/m² were 1.60 minutes (95% CI 0.56-2.64) at BMI 40 kg/m², 3.38 minutes (95% CI 2.02-4.73) at BMI 45 kg/m², and 5.33 minutes (95% CI 3.37-7.30) at BMI 50 kg/m². Excluding 135 patients linked to confirmed all-day lists left 755 patients across 430 theatre lists. In this lognormal model, the corresponding adjusted mean differences were 1.17 minutes (95% CI 0.16-2.27), 2.81 minutes (95% CI 1.45-4.24), and 4.78 minutes (95% CI 2.60-7.12), respectively.

Residual plots from the lognormal model showed remaining departures from normality in the tails. BMI estimates were similar when individual patients or theatre lists were omitted in turn. For BMI 50 compared with 30 kg/m², the adjusted time ratio ranged from 1.231 to 1.298 when individual lists were omitted, compared with 1.260 in the full model. The estimate for interpreter requirement varied more when individual theatre lists were omitted.

Among the 890 patients included in the adjusted analysis, 226 had an interval ≥22 minutes. In mixed-effects logistic regression, BMI was associated with prolonged intervals (overall likelihood-ratio χ²=19.96; df=3; p<0.001), although evidence of nonlinearity was uncertain (χ²=5.80; df=2; p=0.055). Compared with BMI 30 kg/m², adjusted ORs were 1.93 (95% CI 1.28-2.90) at BMI 40 kg/m², 2.95 (95% CI 1.78-4.88) at BMI 45 kg/m², and 4.57 (95% CI 2.22-9.42) at BMI 50 kg/m².

Afternoon sessions were associated with lower odds of prolonged intervals than morning sessions (adjusted OR 0.55; 95% CI 0.37-0.82; p=0.003). The 95% CIs included 1 for age, the ASA comparisons, site, mixed-list status, documented history of difficult airway, obstructive sleep apnoea or severe respiratory disease, severe anxiety or needle phobia, and interpreter requirement.

List-level sample

Among the 611 elective gynaecology lists, 302 (49.4%) overran. Mixed lists accounted for 455 (74.5%), and 196 (32.1%) had a high prolonged-interval burden. Lists contained a median (IQR) of 3 (2-3) cases and a mean (standard deviation) of 2.8 (1.1), with a range of 1-7. There were 193 (31.6%) morning sessions, 349 (57.1%) afternoon sessions, and 69 (11.3%) all-day sessions. Of these lists, 292 (47.8%) were performed at QEH and 319 (52.2%) at UHL.

Descriptive overrun patterns

Overrun occurred in 255/455 (56%) mixed lists and 47/156 (30.1%) lists containing only minor procedures. Among lists with a high prolonged-interval burden, 111/196 (56.6%) overran, compared with 191/415 (46%) lists with a lower burden. Overrun occurred in 137/193 (71%) morning sessions, 134/349 (38.4%) afternoon sessions, and 31/69 (44.9%) all-day sessions. The corresponding rates by site were 176/319 (55.2%) at UHL and 126/292 (43.2%) at QEH.

Multivariable analysis of schedule overrun

In the adjusted list-level model (n=611), mixed lists had higher odds of overrun than lists containing only minor procedures (adjusted OR 3.10; 95% CI 2.00-4.80; p<0.001). A high prolonged-interval burden was also associated with higher odds of overrun (OR 1.52; 95% CI 1.04-2.23; p=0.032). Compared with morning sessions, odds of overrun were lower for afternoon sessions (OR 0.23; 95% CI 0.15-0.34; p<0.001) and all-day sessions (OR 0.18; 95% CI 0.09-0.37; p<0.001). Odds were higher at UHL than at QEH (OR 2.18; 95% CI 1.49-3.20; p<0.001). There was no clear evidence of an association with total case number (OR per additional case 1.20; 95% CI 0.97-1.48; p=0.102). The overall model was statistically significant (likelihood-ratio χ²=106.18; df=6; p<0.001), with McFadden's R² of 0.125, Nagelkerke's R² of 0.213, and AUC of 0.718 (Table 3).

**Table 3:** Multivariable logistic regression analysis of factors associated with theatre list overrun The analysis included 611 elective gynaecology lists containing at least one minor procedure; 302 lists (49.4%) overran. Overrun was defined as the last patient leaving theatre after the scheduled list finish. All predictors shown were included together in the model. A mixed list contained both minor and major procedures. High prolonged-interval burden was defined as ≥50% of minor procedures having an interval from anaesthetic start to surgery start of ≥22 minutes; the reference category was <50%. This measure was calculated retrospectively from recorded timings and was unavailable when lists were scheduled. CIs and two-sided p-values were calculated using Wald methods. OR: odds ratio; CI: confidence interval; UHL: University Hospital Lewisham; QEH: Queen Elizabeth Hospital

Comparison	Adjusted OR	95% CI	P-value
Mixed list versus list containing only minor procedures	3.10	2.00-4.80	<0.001
High versus lower prolonged-interval burden	1.52	1.04-2.23	0.032
Total case number, per additional case	1.20	0.97-1.48	0.102
Afternoon versus morning session	0.23	0.15-0.34	<0.001
All-day versus morning session	0.18	0.09-0.37	<0.001
UHL versus QEH	2.18	1.49-3.20	<0.001

Sensitivity analysis excluding all-day lists

Excluding 69 all-day lists left 542 lists for sensitivity analysis. Mixed lists remained associated with higher odds of overrun (adjusted OR 3.28; 95% CI 2.08-5.18; p<0.001). Odds were also higher at UHL than at QEH (OR 2.50; 95% CI 1.65-3.79; p<0.001), while afternoon lists had lower odds than morning lists (OR 0.23; 95% CI 0.15-0.35; p<0.001). Evidence for an association with high prolonged-interval burden was uncertain (OR 1.40; 95% CI 0.92-2.11; p=0.114). Each additional case was associated with higher odds of overrun (OR 1.43; 95% CI 1.10-1.85; p=0.007). The overall model was statistically significant (likelihood-ratio χ²=106.68; df=5; p<0.001), with McFadden's R² of 0.142, Nagelkerke's R² of 0.238, and AUC of 0.730 (Table 4).

**Table 4:** Sensitivity analysis of factors associated with theatre list overrun after excluding all-day lists After excluding 69 all-day lists, the analysis included 542 elective gynaecology lists containing at least one minor procedure; 271 lists (50%) overran. Overrun was defined as the last patient leaving theatre after the scheduled list finish. All predictors shown were included together in the multivariable logistic regression model. A mixed list contained both minor and major procedures. High prolonged-interval burden was defined as ≥50% of minor procedures having an interval from anaesthetic start to surgery start of ≥22 minutes; the reference category was <50%. This measure was calculated retrospectively from recorded timings and was unavailable when lists were scheduled. CIs and two-sided p-values were calculated using Wald methods. OR: odds ratio; CI: confidence interval; UHL: University Hospital Lewisham; QEH: Queen Elizabeth Hospital

Comparison	Adjusted OR	95% CI	P-value
Mixed list versus list containing only minor procedures	3.28	2.08-5.18	<0.001
High versus lower prolonged-interval burden	1.40	0.92-2.11	0.114
Total case number, per additional case	1.43	1.10-1.85	0.007
Afternoon versus morning session	0.23	0.15-0.35	<0.001
UHL versus QEH	2.50	1.65-3.79	<0.001

Cancellations

Of 231 recorded cancellations, 90 (39%) were attributed to list overruns. Inaccurate date recordings prevented deterministic linkage to specific theatre lists; cancellation data were therefore limited to descriptive summaries, and associations with list-level factors could not be formally evaluated.

## Discussion

In this retrospective study across two hospitals, BMI was nonlinearly associated with the interval from anaesthetic start to surgery start in hysteroscopy. Compared with BMI 30 kg/m², adjusted mean intervals were approximately 1.4 minutes longer at BMI 40 kg/m² and 4.7 minutes longer at BMI 50 kg/m². These BMI findings were similar in analyses using untransformed duration and excluding patients linked to all-day lists. At the list level, mixed lists and lists at UHL had higher odds of overrun, while afternoon lists had lower odds than morning lists. These associations were observed in both list-level models. Evidence for an association between high prolonged-interval burden and overrun was less certain after excluding all-day lists.

The association between BMI and duration may partly reflect additional preparation requirements. Two obstetric cohort studies reported longer intervals from anaesthetic start to surgery start at higher BMI, with one demonstrating that the association persisted after adjustment [14,15]. Differences in patients, procedures, and anaesthetic practice limit direct comparison with hysteroscopy. Additional requirements for transfer, positioning, and vascular access are possible contributors [16]. However, these tasks were not timed separately in our study, and anaesthetic technique was not included in the models, so we cannot determine their contribution. A large elective surgical cohort found no evidence of a further increase in the odds of multiple intubation attempts above BMI 30 kg/m² [17]. Our findings therefore cannot be attributed specifically to difficult intubation and do not establish a BMI threshold or a fixed additional booking time.

Analysing duration continuously avoided the information loss caused by classifying intervals only as prolonged or not prolonged [18]. However, findings for other pre-assessment characteristics were less consistent. The association between ASA physical status III and longer duration was less certain after excluding all-day lists, and estimates for several uncommon characteristics had wide confidence intervals. The binary fields provided limited detail about severity and preparation requirements. They also relied exclusively on explicit documentation in nursing pre-assessment records. Undocumented findings were coded 0, meaning that this category combined documented absence with non-documentation. Incomplete recording of comorbidities in preoperative assessments has also been reported elsewhere [19]. These limitations mean that uncertain associations should not be interpreted as evidence that the characteristics are unimportant for anaesthetic preparation.

Afternoon sessions were associated with shorter intervals in the primary duration model and lower odds of prolonged intervals in the secondary analysis. After excluding patients linked to all-day lists, the estimated duration association was similar, but its confidence interval included 1 (time ratio 0.939; 95% CI 0.878-1.004). Among 44,418 congenital cardiac procedures, afternoon starts were also associated with a 4.2-minute shorter anaesthesia-ready time [20]. However, differences in patient populations and outcome definitions limit direct comparison with hysteroscopy. Possible explanations for our findings include differences in case selection, case order, and the readiness of teams and equipment. These factors were not measured, so the observed association cannot establish a causal effect of session timing.

Mixed list composition was associated with higher odds of overrun in both list-level analyses. The association with total case number was uncertain in the full cohort, but more cases were associated with higher odds of overrun after excluding all-day lists. These findings suggest that both list composition and case number merit consideration when examining theatre list overrun.

Previous studies have used procedure-specific durations and their variability to estimate workload and plan operating lists [21,22]. In one observational study, switching between procedure types was associated with 6.48% longer operative duration, whereas repetition was associated with shorter duration [23]. Anaesthetic technique has also been associated with variation in procedure duration [24]. Delays can occur at several stages of the theatre pathway, and late starts do not necessarily lead to late finishes [25,26]. These findings support evaluating procedure mix and expected duration alongside case number when planning lists. However, our observational findings do not establish that mixed composition causes overrun or that separating minor and major procedures would improve list completion.

The association between high prolonged-interval burden and overrun was less certain after excluding all-day lists. This measure was calculated from intervals observed during the list and was unavailable when the list was booked. Its association with overrun may partly reflect longer intervals contributing to overall list duration. Furthermore, the 22-minute threshold was derived from hysteroscopy and may not be equally appropriate for all minor procedures. On lists containing only one minor procedure, the classification depended entirely on that case. Future studies should evaluate whether information available before list finalisation can predict overrun and improve scheduling.

Associations between overrun and both site and session may reflect local staffing, theatre layout, recovery capacity, sequencing, case mix, booking practices, or other processes. Because these mechanisms were unmeasured, site and session may act as proxies for organisational factors rather than causal determinants; findings are context-specific and hypothesis-generating.

These findings support testing whether anticipated preparation requirements and list composition can improve booking estimates. Previous research has evaluated explicit allocation of anaesthesia time [27], while Getting It Right First Time (GIRFT) guidance recommends reviewing case order, staffing, equipment, and postoperative requirements before surgery [28]. Structured recording of anticipated positioning, vascular access, airway management, and other logistical requirements could provide additional information for scheduling. BMI could be considered alongside these factors, but the adjusted mean differences reported here are not validated booking allowances for individual patients. Any new approach, including changes to mixed-list composition, should use information available before list finalisation and undergo prospective evaluation of overruns, cancellations, patient flow, and safety.

Strengths include routinely collected data from two hospital sites, structured data extraction, and complementary patient- and list-level analyses. The primary analysis retained duration as a continuous outcome and accounted for patients sharing the same theatre list. The model allowed a nonlinear association with BMI, and adjusted mean differences were reported in minutes. Sensitivity analyses examined untransformed duration and the exclusion of all-day lists, while influence analyses assessed the contribution of individual patients and lists. Missing data and the handling of undocumented pre-assessment findings were described explicitly.

The retrospective design limits causal interpretation. The primary continuous analysis was introduced after the original binary analyses, and the lognormal model was selected after reviewing residual plots; these analyses should therefore be considered exploratory. Pre-assessment characteristics relied exclusively on nursing records, and coding undocumented findings as 0 may have underestimated their frequency. Estimates at very high BMI were uncertain because few patients had these values, and the findings do not establish a BMI threshold or booking rule. The ≥22-minute threshold used in secondary analyses was derived from this cohort and has not been externally validated. High prolonged-interval burden was calculated retrospectively and was unavailable when lists were scheduled. Complete-case analysis excluded 23 of 913 patients (2.5%), which may have introduced selection bias. Unmeasured differences in anaesthetic technique, staffing, case order, equipment, and other workflow factors may also have influenced the observed associations. Finally, findings from two hospitals within one NHS trust may not generalise to other settings.

Although the patient-level models accounted for theatre-list clustering, list linkage was provisional, and errors in assigning patients to lists remain possible. Many lists contributed only one hysteroscopy, providing limited information about variation within those lists. Residual plots showed remaining departures from normality, and the bootstrap confidence intervals depended on the assumptions of the fitted lognormal model. Although the BMI findings were similar in sensitivity analyses, these checks do not remove all uncertainty about model assumptions. Separately, 90 of 231 cancellations were attributed to overrun, but unreliable linkage to individual theatre lists prevented assessment of whether the identified list-level factors were associated with cancellations.

Future studies should evaluate these findings in other hospitals and develop and validate scheduling models using information available before theatre lists are finalised. Prospective studies should then test whether incorporating expected preparation time and list composition into booking reduces overruns and cancellations while assessing patient safety, staff workload, and patient flow.

## Conclusions

In this retrospective study, BMI was nonlinearly associated with the interval from anaesthetic start to surgery start in hysteroscopy, with longer adjusted mean intervals at higher BMI values. Lists combining minor and major procedures had higher odds of overrun than lists containing only minor procedures. Odds of overrun were also higher at UHL than at QEH and lower for afternoon than morning lists. These findings support prospective evaluation of preparation requirements and list composition when planning theatre lists. They do not establish causal relationships, a BMI threshold, or a validated booking rule.
